# 1884. Metagenomic Sequencing of the Lung Microbiota Facilitates Diagnosis and Prognosis of Nontuberculous Mycobacterial Pulmonary Disease

**DOI:** 10.1093/ofid/ofad500.1712

**Published:** 2023-11-27

**Authors:** Yu Chen, Qing Miao, Bijie Hu

**Affiliations:** Department of Infectious Diseases, Zhongshan Hospital, Fudan University, Shanghai, Shanghai, China; Department of Infectious Diseases, Zhongshan Hospital, Fudan University, Shanghai, Shanghai, China; Department of Infectious Diseases, Zhongshan Hospital, Fudan University, Shanghai, Shanghai, China

## Abstract

**Background:**

The incidence of nontuberculous mycobacterial pulmonary disease (PNTM) is rising, but available diagnostics and treatments have limitations. The lung microbiota plays an important role in the onset and progression of lung diseases. Thus, investigating the relationship between the lung microbiota and treatment outcomes in PNTM should contribute to the development of improved therapeutic, diagnostic, and prognosis-determination tools.

**Methods:**

Bronchoalveolar lavage fluid (BALF) was collected from 169 patients with PNTM, 46 patients with pulmonary tuberculosis (PTB), 17 individuals without mycobacterial infection, and 37 individuals without infection. The lung microbiota in BALF was characterized using metagenomic sequencing. Patients with PNTM and PTB were identified with a microbiome-based classifier. Different pneumotypes were defined and associated with outcomes.

**Results:**

Analysis of BALF samples revealed that patients with PNTM differed from controls in terms of lung microbiota richness and composition. The species co-occurrence network in PNTM exhibited low diversity and predominantly positive interactions. Random forest models improved the classification efficacy of PNTM and PTB based on the lung microbiota. At the 13-month median follow-up, pneumotype 1 (with *Mycobacterium*, opportunistic pathogens, and anaerobes) had a lower probability of sustained culture conversion (hazard ratio = 0.29; 95% confidence interval = 0.11–0.72; P = 0.007) than pneumotype 2, indicating a worse prognosis .

Differences in lung microbial taxonomic characteristics between PNTM patients and PTB patients.

**Figure:**
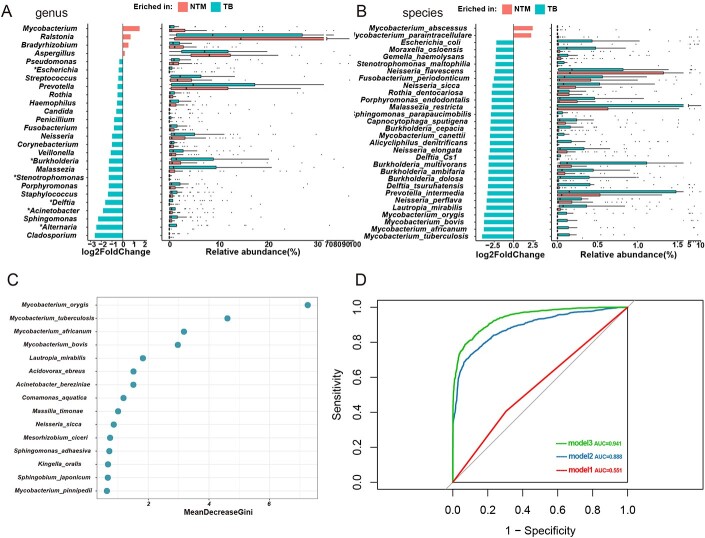

(A and B) Relative abundance comparisons at the genus (A) and species (B) levels including major bacteria and fungi. In the histogram, microbial taxa that were overrepresented in the PNTM patients were depicted on the right (log2foldchange >0), and microbial taxa that were overrepresented in the PTB patients were depicted on the left (log2foldchange <0). Boxes indicate the horizontal line in each box represents the median, the top and bottom of the box the 25th and 75th percentiles, and the whiskers 1.5 times the interquartile range. (C and D) Classification of PNTM and PTB by Random Forest Model. (C) The top 15 most discriminating taxonomic group between PNTM and PTB. (D) The performance of classifiers in the cohort was measured by the area under the ROC curve (AUC). Cross-validation AUCs based on 5-fold cross-validation replicates of 10 were provided for microbiota classifiers. Model 1 contained mNGS diagnostic criteria. Model 2 contained all differential species. Model3 contained important species filtered by recursive feature elimination (RFE). Hypothesis testing (A) P-values were determined using the Mann-Whitney U test, and the Benjamini-Hochberg was applied to adjust p values. Significant taxa differences (adjusted P-value<0.05) were observed as *. (B) Use the DESeq2 package to calculate log2foldchange. All species displayed were different at the level of p < 0.05. PNTM, nontuberculous mycobacterial pulmonary disease; PTB, pulmonary tuberculosis; mNGS, metagenomic next-generation sequencing.

Pneumotype can predict the microbial outcome of PNTM.

**Figure:**
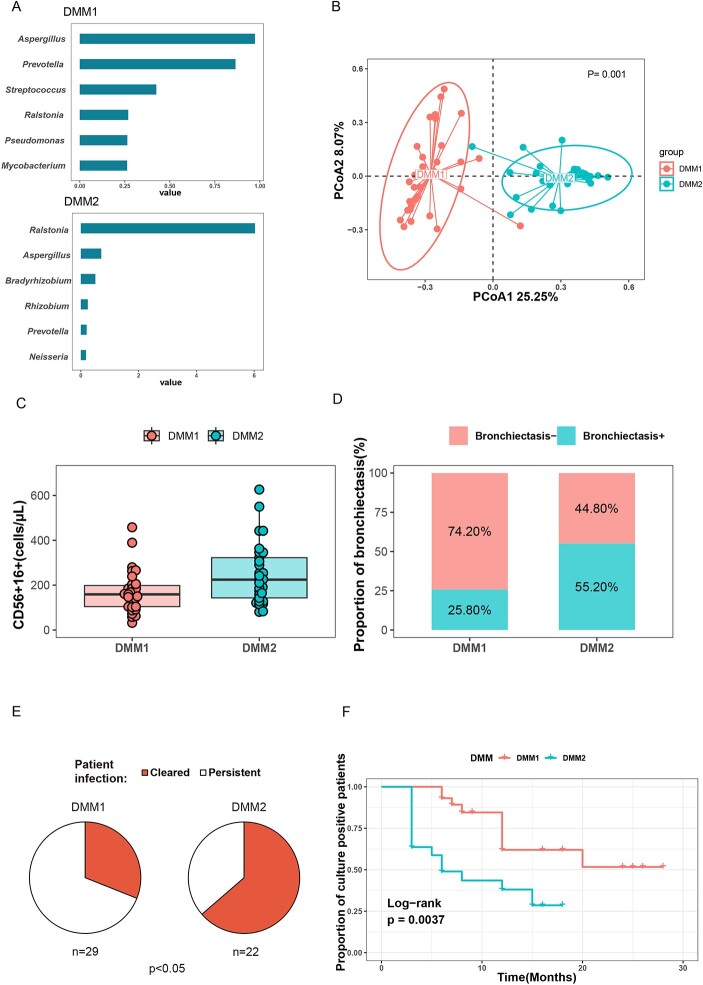

(A) Dirichlet Multinomial Mixtures (DMM) identified 2 compositionally distinct microbial communities of PNTM patients. The proportion of communities in each microbial state at the genus level. (B) Principal component analysis of microbiota communities revealed that the community composition of lung microbiota was dis-tinct (PERMANOVA, R2=0.14, P=0.001). (C) Significant differences were found be-tween DMM1 and DMM2 in terms of CD56+16+ counts (p=0.035). (D) The proportion of bronchiectasis was significantly different between DMM1 and DMM2 (p=0.02). (E) Cultures conversion rate was significantly greater in patients with DMM2 pneumotype than those with DMM1 pneumotype. (F) Kaplan–Meier curves of persistent positive cultures during follow-up in patients stratified by pneumotype. Hypothesis testing was performed using the (C) Mann-Whitney U test, (D and E) χ2 test, (F) Log-rank test. PNTM, nontuberculous mycobacterial pulmonary disease; PTB, pulmonary tuberculosis.

**Figure:**
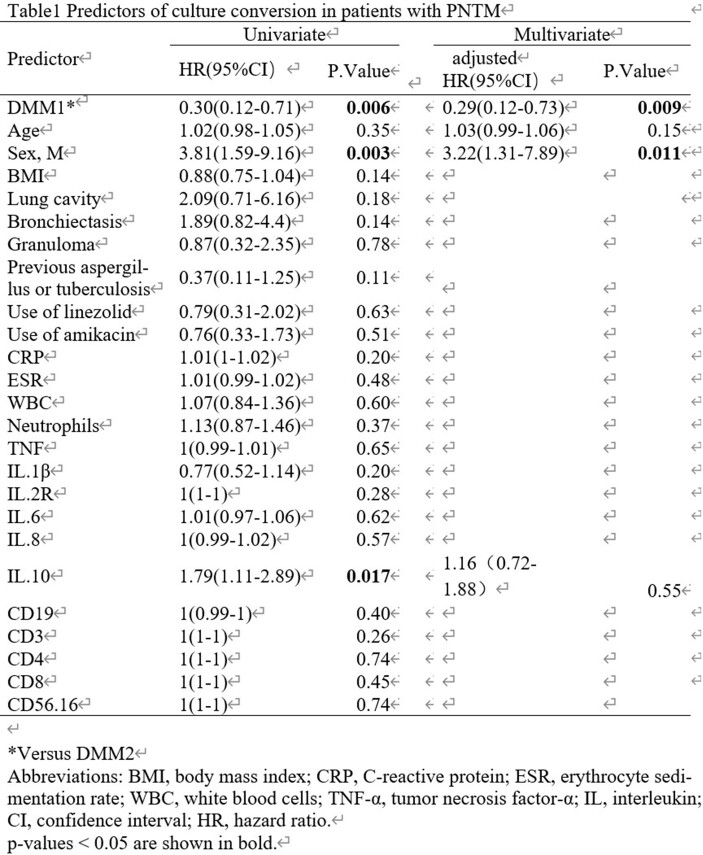

**Conclusion:**

Characterization of the lung microbiota improved diagnostic efficacy and identified high-risk patients. We conclude that sampling the lung microbiota can aid in clinical decision-making and provide novel therapeutic avenues for PNTM.

**Disclosures:**

**All Authors**: No reported disclosures

